# Amygdala gene alterations in Parkinson's disease

**DOI:** 10.1002/alz70855_106020

**Published:** 2025-12-24

**Authors:** Cécilia Tremblay, Gabriella Frosi, Sidra Aslam, Jessica Walker, Anthony J Intorcia, Parichita Choudhury, Charles Adler, Holly Shill, Shyamal Mehta, Corina Nagy, Erika Driver‐Dunckley, Alireza Atri, Thomas G Beach, Geidy E Serrano, Yashar Zeighami

**Affiliations:** ^1^ Douglas Research Center, Montreal, QC, Canada; ^2^ Mcgill university, Montreal, QC, Canada; ^3^ Banner Sun Health Research Institute, Phoenix, AZ, USA; ^4^ Banner Sun Health Research Instittue, Sun City, AZ, USA; ^5^ Banner Sun Health Research Institute, Sun City, AZ, USA; ^6^ Parkinson's Disease and Movement Disorders Center, Mayo Clinic, Scottsdale, AZ, USA; ^7^ Barrow Neurological Institute, Phoenix, AZ, USA; ^8^ Mayo Clinic Arizona, Scottsdale, AZ, USA; ^9^ Douglas Mental Health University Institute, Montréal, QC, Canada; ^10^ Montreal Neurological Institute, McGill University, Montréal, QC, Canada

## Abstract

**Background:**

The amygdala is closely connected to the olfactory bulb and involved in olfactory processing as well. It is a particularly susceptible region that accumulates a‐synuclein pathology in Parkinson's disease (PD). Our previous work revealed olfactory bulb gene alterations related to neuroinflammation, and neurotransmitter dysfunction to be associated with olfactory function. We aimed to extend this work and assess gene expression changes, affected pathways and co‐expression networks by transcriptomic profiling of the amygdala in subjects with and without clinicopathologically defined PD.

**Method:**

Bulk RNA sequencing was performed on frozen human amygdala of 20 PD and 20 controls without dementia or any other neurodegenerative disorder, from the Arizona Study of Aging and Neurodegenerative Disorders.

**Result:**

Differential expression analysis, corrected for age, sex and post‐mortem interval revealed 40 significantly differentially expressed genes (DEGs) in PD. Downregulated genes were mainly related to altered hemoglobin expression while upregulated genes included inflammatory markers and glutamine transporter. Significantly enriched pathways were involved in oxygen and carbon dioxide transport, cellular oxidant detoxification and regulation of glutamine secretion. Cell enrichment revealed these genes to be mainly expressed in neurons. Co‐expression network analysis using *Weighted correlation network analysis (WGCNA*) subsequently identified four significant modules correlated with both PD and premortem olfactory dysfunction which were involved in pathways related to neuroinflammatory processes, mitochondrial, endoplasmic reticulum and lysosomal dysfunction.

**Conclusion:**

These preliminary results suggest cellular alterations in the amygdala related to mitochondrial dysfunction, altered oxygen homeostasis, oxidative stress, inflammation as well as glutamatergic neurotransmitter dysfunction that may contribute to neurodegeneration in PD.

